# Chronic Obstructive Pancreatitis as a Delayed Complication of Pancreatic Trauma

**DOI:** 10.1155/1991/73834

**Published:** 1991

**Authors:** Edward L. Bradley

**Affiliations:** 1 Piedmont Professor of Surgery Emory University Atlanta Georgia USA; 2 Department of Surgery 1968 Peachtree Road N.W. Atlanta GA 30309 USA

## Abstract

Increasing surgical experience with the immediate consequences of pancreatic injuries has resulted from parallel growth in the volume of motor vehicle accidents and societal violence. However, few surgeons are aware that complications may be considerably delayed following pancreatic trauma, occurring in some cases months to years after apparent recovery from the original injury. In four patients with blunt pancreatic trauma initially treated by non-operative means, stricture of the main pancreatic duct developed over a period of months as a result of progressive fibrosis at the site of ductal injury. Pancreatic duct hypertension was demonstrated to be present in the obstructed duct, and secondary changes of chronic pancreatitis developed in the obstructed segment of the gland (“upstream” chronic pancreatitis). Seven similar patients with delayed onset of chronic obstructive pancreatitis after pancreatic trauma were found in the literature. Symptoms related to these acquired ductal strictures are most commonly those of abdominal pain and recurrent episodes of acute pancreatitis. Recognition of post-traumatic chronic obstructive pancreatitis principally involves awareness that injuries to the pancreatic duct can produce remote complications. Pancreatoenteric drainage, or resection of the obstructed segment of pancreas, provides prompt and effective relief.


*HPB Surgery*, 1991, Vol. 5, pp. 49–60
Reprints available directly from the publisher
Photocopying permitted by license only

© 1991 Harwood Academic Publishers GmbH
Printed in the United Kingdom

### CASE REPORT

### CHRONIC OBSTRUCTIVE PANCREATITIS AS A DELAYED COMPLICATION OF PANCREATIC TRAUMA

EDWARD L. BRADLEY, III
*Piedmont Professor of Surgery, Emory University, Atlanta, Georgia, USA*

*(Received 13 May 1991)*

Increasing surgical experience with the *immediate* consequences of pancreatic injuries has resulted from parallel growth in the volume of motor vehicle accidents and societal violence. However, few surgeons are aware that complications may be considerably delayed following pancreatic trauma, occurring in some cases months to years after apparent recovery from the original injury. In four patients with blunt pancreatic trauma initially treated by non-operative means, stricture of the main pancreatic duct developed over a period of months as a result of progressive fibrosis at the site of ductal injury. Pancreatic duct hypertension was demonstrated to be present in the obstructed duct, and secondary changes of chronic pancreatitis developed in the obstructed segment of the gland ("upstream" chronic pancreatitis). Seven similar patients with delayed onset of chronic obstructive pancreatitis after pancreatic trauma were found in the literature. Symptoms related to these acquired ductal strictures are most commonly those of abdominal pain and recurrent episodes of acute pancreatitis. Recognition of post-traumatic chronic obstructive pancreatitis principally involves awareness that injuries to the pancreatic duct can produce remote complications. Pancreatoenteric drainage, or resection of the obstructed segment of pancreas, provides prompt and effective relief.

KEY WORDS: Pancreatic trauma, chronic pancreatitis, pancreatic duct stenosis

As a result of the rising toll of motor vehicle accidents and violent crimes, surgical experience with pancreatic trauma has dramatically increased. Since most penetrating abdominal wounds are explored without delay, experience with the immediate surgical management of open pancreatic injuries has been extensive[1,2].

Blunt pancreatic injuries, on the other hand, occur less commonly, and can be difficult to diagnose. As a result, the management of patients with suspected blunt pancreatic trauma has been controversial. Several groups have recommended an initial non-surgical approach to selected patients with suspected pancreatic injuries from blunt trauma[3–5]. Others have advocated immediate surgical exploration in order to minimize morbidity and mortality[6].

Recently, while in the process of a long term evaluation of selective non-operative management of blunt pancreatic trauma, we discovered that a number of patients had developed complications of blunt pancreatic trauma several months following apparent recovery from the effects of the original injury. In each of these

Address correspondence to: E.L. Bradley, III, M.D., Department of Surgery, 1968 Peachtree Road, N.W. Atlanta, GA 30309, USA.

patients, progressive fibrosis at the site of an uncorrected injury to the main pancreatic duct had resulted in stricture of the duct with proximal dilatation. All patients became symptomatic, and chronic pancreatitis was demonstrated to have developed in the obstructed segment of pancreas. Despite an extensive search of the literature, only three previous references have cited patients developing chronic pancreatitis as a result of ductal stricture after pancreatic trauma[6–8]. Accordingly, the purposes of this report are threefold; to make the surgical community more aware of this delayed complication of pancreatic trauma, to describe the clinical circumstances under which this diagnosis should be considered, and to suggest how this post-traumatic obstructed duct syndrome should be managed.

### PATIENTS AND METHODS

Although prompt surgical exploration for all patients with penetrating abdominal injuries has been a longstanding departmental policy at Emory, in the absence of peritoneal signs, non-operative management has frequently been selected for patients with blunt abdominal trauma. Seventeen patients with blunt abdominal trauma and suspected pancreatic injury were chosen for non-operative management at Emory University between 1985–1990.

In this highly selected group, there were 11 males and 6 females, with an age range of 2–67 years. Pancreatic trauma was suspected on the basis of: persistent hyperamylasemia (15/17) (avg. peak amylase 1325 IU), abnormal pancreatic morphology on CT (12/17) (pseudocyst [7], pancreatic enlargement [4], pancreatic fracture [1]), subsequent findings at surgery (3 patients), and abnormal endoscopic pancreatography (3 cases).

During an average follow-up of 12 months, four of these 17 patients required re-admission for symptoms of persistent abdominal pain or recurrent episodes of acute pancreatitis. A pancreatic duct obstruction was found in each of the four patients. This complication occurred 2–10 months following recovery from the original injury, and is the subject of this report.

### CASE I

A 25 year old white female, non-drinker, was involved in an all-terrain vehicle accident 09/04/90, sustaining a handlebar injury to the epigastrium. A pancreatic injury was suspected, but not confirmed during exploration at another hospital. Post-operatively, the patient began to drain large volumes of pancreatic juice through a drain placed at surgery. Following transfer to our hospital on 10/09/90, an ERCP demonstrated a ruptured main pancreatic duct in the head of the pancreas (Figure 1). Somatostatin analog, 200mg SQ q 8 hours, resulted in a decrease in fistula output from 1000cc/day to 200cc/day, and the patient was discharged on oral feedings.

She was re-admitted on 11/06/89, with weakness, weight loss, and abnormal liver function tests (total bilirubin 3.9, alkaline phosphatase 198, SGOT 527, gamma GT 153). A diagnosis of cholestasis was made. After discontinuing the somatostatin, liver function normalized, and the patient was once again discharged.

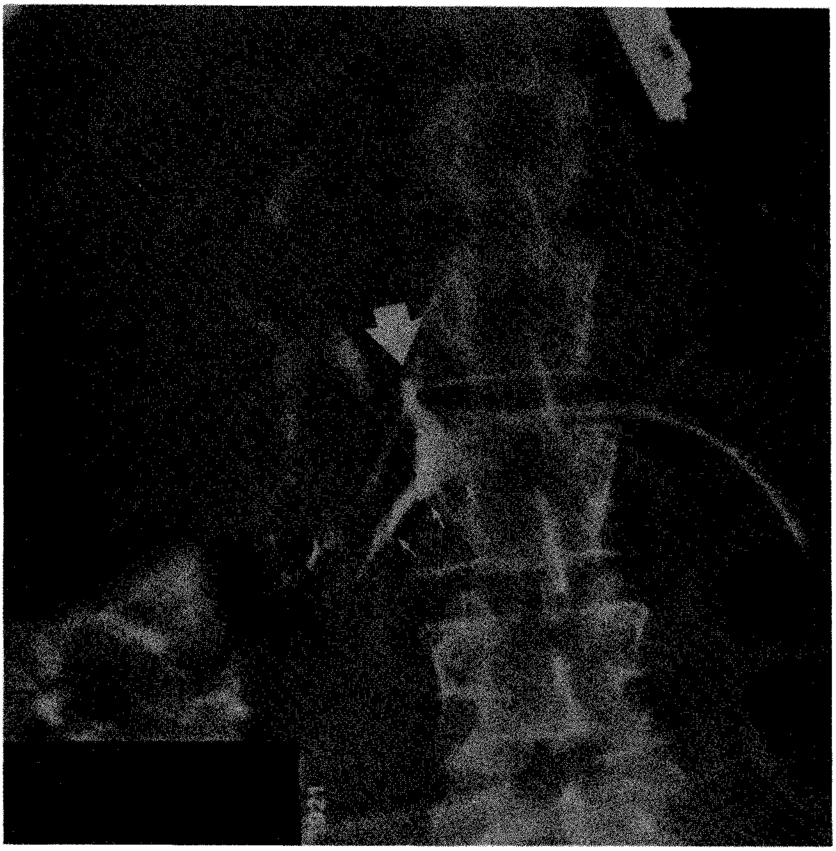

**Figure 1** ERCP obtained 4 weeks after original injury. Note proximal main pancreatic duct and site of ductal injury (large arrow). Pancreatic juice collection (small arrows) is being controlled by drain placed at original operation.

At home, however, she began to experience repeated episodes of acute abdominal pain associated with nausea and vomiting. On 01/12/90, she was re-admitted with a particularly severe episode of abdominal pain, which was accompanied by a serum amylase of 1200 IU. A CT scan demonstrated pancreatic duct dilation, beginning at the fistula site (Figure 2). At surgery on 01/28/90, the head of the pancreas appeared normal, but the distal pancreas exhibited the typical morphologic changes of chronic pancreatitis. Pressure in the obstructed distal pancreatic duct was markedly elevated (42cm $H_2O$). A longitudinal pancreatojejunostomy Roux-en-Y, incorporating the dilated distal pancreatic duct, as well as the normal pancreatic duct cephalad to the area of duct disruption, was performed (Figure 3). A biopsy of the distal fibrotic pancreas was returned as "severe chronic pancreatitis". She has been asymptomatic for the past 15 months.

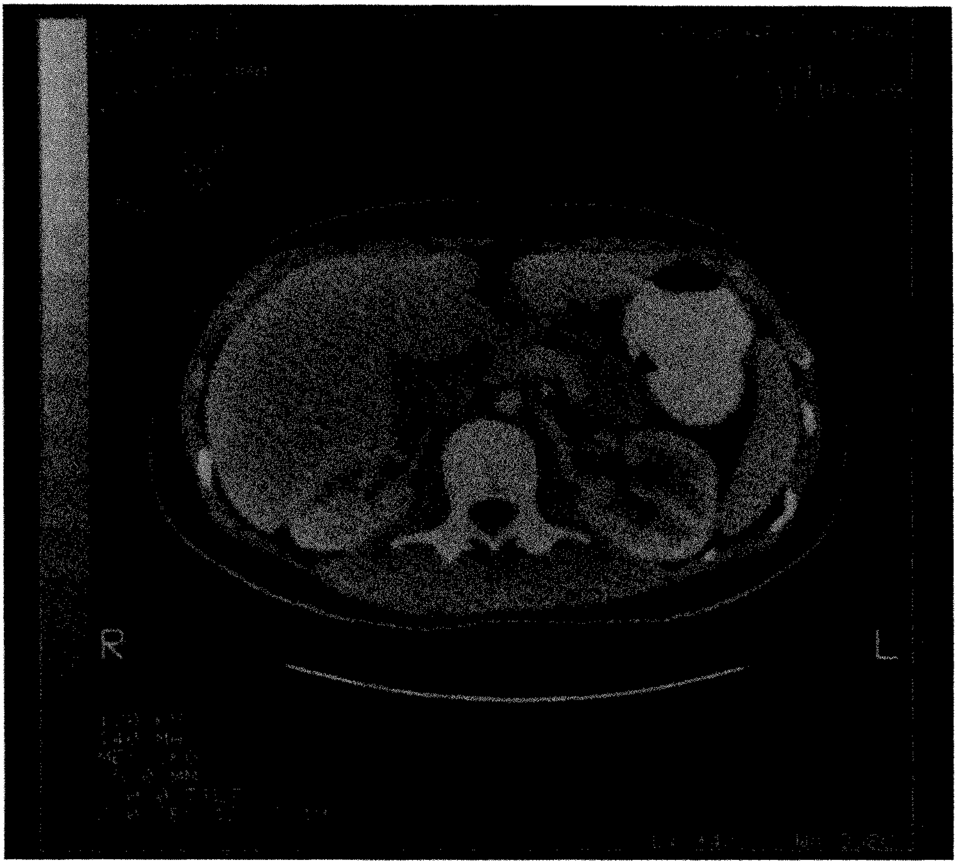

**Figure 2** CT scan demonstrating dilatation of the caudal portion of the main pancreatic duct (arrows) resulting from traumatic transection of the gland 3 months previously.

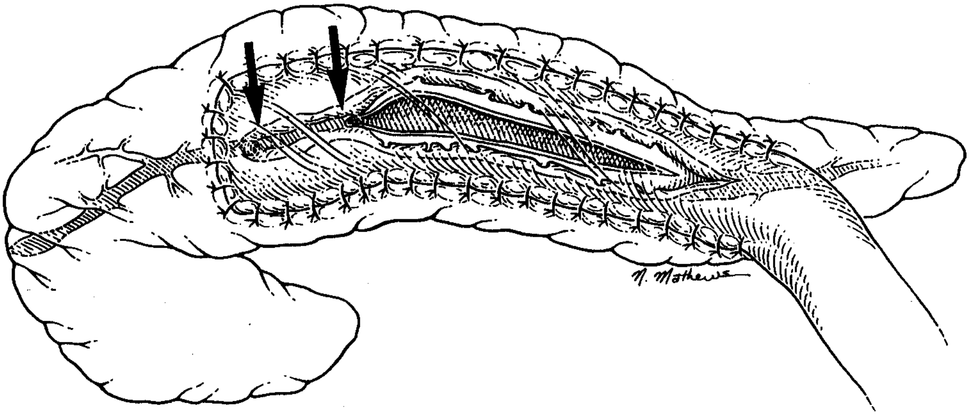

**Figure 3** Artist's rendition of the surgical procedure performed in Case I. Note the width of the scar (arrows) with segmental loss of the main pancreatic duct resulting from the previous traumatic transection. Note also the normal cephalic portion of the duct, and the dilated duct proximal to the region of fibrosis.

### CASE II

A 14 year old male honor student suffered a bicycle handle bar injury to the epigastrium on 08/14/87. As no rebound was present, an expectant course was chosen despite serum amylase rising from 320 IU to 3000 IU during the first week. A subsequent CT scan demonstrated a 5cm pseudocyst in the mid-body of the pancreas. A two week trial of transcutaneous drainage failed to resolve the pseudocyst, and a Roux-en-Y cystojejunostomy was performed on 09/24/87. In spite of complete resolution of the pseudocyst, all attempts to feed the patient resulted in increased abdominal pain and renewed hyperamylasemia. An attempted ERCP was unsuccessful.

At re-exploration on 11/12/87, the head and neck of the pancreas was normal to observation and palpation, while the distal pancreas was hard and fibrotic. During the performance of a distal pancreatectomy, the pancreatic duct distal to the Roux-en-Y insertion was noted to be markedly dilated and under pressure (50 cm $H_2O$). Histologic examination of the resected distal pancreas revealed "typical changes of chronic pancreatitis" (Figure 4). The post-operative course was unremarkable. The patient has remained pain free since discharge.

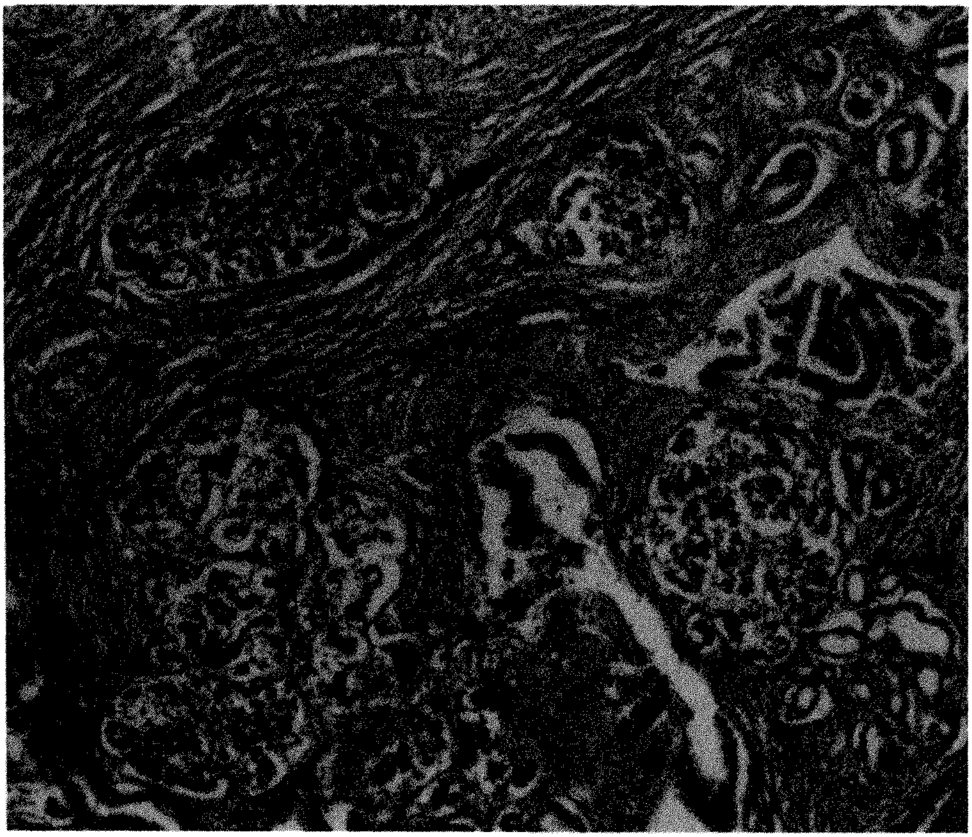

**Figure 4** Photomicrograph of the resected pancreas proximal to a post-traumatic delayed duct obstruction in a 14 year old. These typical changes of chronic pancreatitis developed within months after the original injury.

### CASE III

A six year old female sustained blunt abdominal trauma as a restrained passenger in a head-on motor vehicle collision on 12/06/89. Abdominal tenderness was present, as were active bowel sounds and a large left upper quadrant ecchymosis. Admission serum amylase was 1000 IU. A subsequent CT scan demonstrated a small liver fracture in the right lobe and an enlarged pancreas. The initial management was non-operative, and while the patient gradually improved, the serum amylase did not. An ERCP on 12/15/89 failed to fill the distal third of the pancreas. A CT scan the following day demonstrated an 8cm pseudocyst arising from the distal pancreas. Transcutaneous external drainage of the pseudocyst was successfully performed.

Two months later, however, the patient was re-admitted for increasing abdominal pain. At surgery, the distal pancreatic duct was tense and enlarged proximal to the site of an injury in the mid-body of the pancreas. A recurrent pseudocyst in the tail of the pancreas was also demonstrated. Following distal pancreatectomy, the patient has subsequently remained free of abdominal complaints. Histologic findings of the resected pancreas were "compatible with chronic pancreatitis".

### CASE IV

A 40 year old non-alcoholic male sustained blunt abdominal trauma from an automobile steering wheel as an unrestrained driver in a motor vehicle accident on 03/09/86. Serum amylase was 750 IU. Generalized peritonitis led to abdominal exploration on the day of admission. Fifteen centimeters of infarcted jejunum were found and resected. The lesser sac was not opened, and the pre-operative hyperamylasemia was attributed to the small bowel injury.

Three months after surgery, however, the patient developed daily abdominal pain, which ultimately became persistent. Due to the abdominal pain, he became anorexic and suffered a 20 lb. weight loss. When seen at our hospital, an ERCP demonstrated a normal proximal pancreatic duct but a stricture in the distal third with upstream dilatation of the duct and delayed emptying of contrast material. He underwent distal pancreatectomy of 08/15/86. Findings at surgery were a normal head and body of the pancreas but a dilated palpable pancreatic duct in a fibrotic distal gland. Permanent section showed "chronic pancreatitis". When last seen 3 months following discharge, the patient was pain free.

### DISCUSSION

Pancreatic duct obstruction can be classified as congenital or acquired. Examples of congenital pancreatic duct obstruction would include various developmental abnormalities of the duct[9,10]. Acquired ductal strictures, on the other hand, most commonly are caused by encroachment of parenchymal tumors[11]. Other examples of acquired ductal strictures occur in alcoholic pancreatitis[12,13], ampullary stenosis[14], and fibrotic stricture after necrotizing pancreatitis[8].

Traumatic injury to the main pancreatic duct ultimately led to fibrosis and duct obstruction at the site of injury in the four patients reported in the current study.

Furthermore, in each one of these cases, morphologically and histologically demonstrated chronic obstructive pancreatitis developed in the segment of pancreas drained by the obstructed duct. This condition has been termed "upstream" chronic pancreatitis[8]. A thorough search of the literature was able to identify only seven previously reported cases in which acquired ductal strictures resulted in chronic obstructive pancreatitis following pancreatic trauma (Table 1)[6–8]. Since it is likely that the incidence of post-traumatic chronic obstructive pancreatitis might increase if non-operative management of suspected pancreatic injuries achieves wider acceptance, it is important that the surgical community at large be made aware that such a complication exists.

In 1971, Bach and Frey described two patients with overlooked traumatic pancreatic transections, who later developed a fibrotic stricture at the site of ductal injury and proximal ductal dilation[6]. In one patient, this was manifested clinically as multiple episodes of recurrent acute pancreatitis, and responded to distal pancreatectomy. The resected specimen was described as "chronic pancreatitis". The other patient expired prior to a planned surgical procedure. Autopsy showed ductal dilation and a "scarred and fibrotic pancreas" proximal to the site of ductal obstruction. Unfortunately, no mention was made of specific histologic findings in either case.

Taxier and his co-workers reported three patients with changes of chronic pancreatitis occurring 3 months to 6 years following blunt trauma to the main pancreatic duct[7]. All three patients had undergone internal drainage of a pancreatic pseudocyst shortly after the original pancreatic injury. In each case, recurrent severe abdominal pain led to re-exploration and the finding of an obstructed main pancreatic duct at the site of pseudocyst drainage. The symptoms responded to distal pancreatectomy in 2 cases, and Roux-en-Y pancreatojejunostomy in the third. Histologic examination of two cases showed "chronic pancreatitis with fibrosis and atrophy" and "marked fibrosis and absence of pancreatic acini".

Laugier *et al.* described the delayed development of chronic obstructive pancreatitis in 2 patients occurring after blunt pancreatic injury[8]. In each case, a traumatic pseudocyst had developed shortly after injury, which was successfully drained. Both of the patients, however, subsequently suffered recurrent bouts of increasing abdominal pain and hyperamylasemia. At re-exploration, the findings were similar in each case: a normal head of the pancreas, dilatation of the main pancreatic duct proximal to a scar in the body of the pancreas, and histologic changes of chronic pancreatitis in the obstructed segment. Symptoms ceased after distal pancreatic resection.

Today, most authorities are in agreement that pancreatic duct obstruction occupies a pivotal position in the initiation of the parenchymal changes characteristic of chronic pancreatitis[12,13]. Precisely how pancreatic duct obstruction is translated into chronic pancreatitis is unknown, but the demonstration of ductal hypertension in the obstructed segment in our trauma patients may be important in this regard. Pancreatic duct hypertension has previously been demonstrated to exist in patients with chronic pancreatitis due to other causes[15].

How long partial or complete obstruction of the pancreatic duct must be present before irreversible changes of chronic pancreatitis develop is not known with certainty. In four of the eleven collected patients, chronic pancreatitis developed in the obstructed segment within four months after the original ductal injury. Austin and his co-workers have shown that even a 75% stenosis of the main pancreatic

**Table 1** Post-traumatic pancreatic duct syndrome

| *Author* | *Sex/Age* | *Initial procedure* | *Indications for re-exploration* | *Second procedure* | *Pancreatic morphology* | *Course* |
|---|---|---|---|---|---|---|
| Bach & Frey, 1971 | M | ED @ 2 days | 1) Recurrent AP<br>2) Persistent Fistula | DP @ 3 months | 1) Transection<br>2) Dilation MPD in caudal segment<br>3) CP in obstructed segment | Well @ 2 years |
| | M | ED @ 1 day | None | None<br>autopsy findings | 1) Transection<br>2) dilatation MPD in caudal segment<br>3) CP in obstructed segment | Expired 2 months after initial procedure |
| Taxier, et al 1980 | F/45 | ID<br>pseudocyst | 1) Recurrent AP | DP @ 6 years | 1) Transection<br>2) CP in obstructed segment* | Well @ 6 months |
| | F/29 | ID<br>pseudocyst | 1) Recurrent AP | DP<br>ID @ 3 years | 1) Transection<br>2) Recurrent pseudocyst<br>3) CP in obstructed segment* | No follow-up |
| | F/27 | ID<br>pseudocyst | 1) Persistent pain | LPJ @ 1 year | 1) Recurrent pseudocyst<br>2) Dilation MPD in caudal segment<br>3) CP in obstructed segment* | Pain free @ 2 months |
| Laugier, et al 1983 | M/19 | ID<br>pseudocyst | 1) Recurrent AP<br>2) Persistent pain | LPJ @ 18 months | 1) Dilatation MPD in caudal segment<br>2) CP in obstructed segment*<br>3) Cranial pancreas normal* | Pain free for 14 years |
| | M/56 | ED | 1) Persistent pain | LPJ<br>ID @ 10 years | 1) Pseudocyst<br>2) Dilatation MPD in caudal segment<br>3) CP in obstructed segment* | Pain free for 7 years |

**Table 1** *continued.*

| *Author* | *Sex/Age* | *Initial procedure* | *Indications for re-exploration* | *Second procedure* | *Pancreatic morphology* | *Course* |
|---|---|---|---|---|---|---|
| Current Study | F/25 | ED @ 1 day | 1) Persistent pain<br>2) Recurrent AP<br>3) Persistent pancreatic fistula | LPJ @ 4 months | 1) Transection<br>2) Dilation MPD in crudal segment<br>3) CP in obstructed segment* | Pain free for 1 year |
| | M/14 | ID pseudocyst @ 10 days | 1) Persistent pain | DP @ 3 months | 1) Transection<br>2) Dilation MPD in caudal segment<br>3) CP in obstructed segment* | Pain free for 1 year |
| | F/6 | ED pseudocyst @ 14 days | 1) Persistent pain | DP @ 2 months | 1) Dilation MPD in caudal segment<br>2) CP in obstructed segment* | Pain free for 1 year |
| | M/40 | Resection infarcted jejunum | 1) Persistent pain | DP @ 5 months | 1) CP in obstructed segment* | Pain free for 3 months |

ED = External drainage

ID = Internal drainage

AP = Acute pancreatitis

MPD = Main pancreatic duct

CP = Chronic pancreatitis

* = Proved by histology

DP = Distal pancreatectomy

LPJ = Longitunidal pancreatojejunostomy duct in cats leads to chronic pancreatitis within 3 months following occlusion[16]. Furthermore, once chronic obstructive pancreatitis had developed, pancreatic function did not recover following release of the occlusion.

Even though traumatic pseudocysts resulting from ductal disruption can be successfully approached by either operative or transcutaneous means, progressive scarring at the site of ductal injury may result in delayed obstruction of the duct. The resultant ductal obstruction, in turn, may lead to symptoms of persistent pain or episodes of recurrent acute pancreatitis in the obstructed segment, and eventually, the development of "upstream" chronic pancreatitis. Since 7 of the 11 collected cases in this report followed drainage of a traumatic pseudocyst arising from the body or tail of the pancreas, it seems reasonable to assume that distal pancreatectomy performed at the initial exploration would not only have prevented obstructive pancreatitis with its attendant symptoms, but also would have obviated the risk and expense of subsequent surgical explorations. Accordingly, if compelling reasons dictate that drainage of a traumatic pseudocyst in the body or tail be chosen over distal resection as primary therapy, careful follow-up is mandatory.

In the absence of any significant long term data concerning pancreatic trauma, the actual incidence of post traumatic chronic obstructive pancreatitis cannot be known with certainty. Moreover, since chronic obstructive pancreatitis has not been widely recognized as a complication of trauma, it is quite possible that the actual incidence may be greater than this initial review of the literature might indicate. From the standpoint of pathophysiology, it seems reasonable to propose that the degree of ductal fibrosis and the subsequent development of post-traumatic delayed duct obstruction might be related to the nature of the pancreatic injury (parenchymal vs ductal), the severity of the ductal injury (circumferential vs lateral), and the form of initial treatment (drainage or resection of the injured segment).

In contrast to patients with injury restricted to the pancreatic parenchyma, the observations in this report serve to reinforce previous demonstrations that significant complications after pancreatic trauma overwhelmingly occur in patients with major ductal injury[2]. For this reason, it seems prudent to evaluate pancreatic ductal integrity by endoscopic pancreatography in all stable patients with suspected pancreatic injury[17]. Pancreatography may be even more important in trauma patients with suspected pancreatic injury who are being considered for non-operative management. Several groups have claimed that endoscopic pancreatography has been successful in distinguishing those patients with ductal injury requiring surgery from those with parenchymal injury who can safely be observed[17-19].

Surgical management of patients with post-traumatic chronic obstructive pancreatitis involves decompression of the obstructed duct by lateral pancreatojejunostomy when dilatation is present, or resection of the involved segment of pancreas when the process is limited to the body or tail and ductal dilatation is not prominent. In order to conserve functional pancreatic tissue, however, patients with strictures in the most cephalic portion of the main pancreatic duct should undergo Roux-en-Y drainage to the obstructed segment of duct.

### *References*

1. Jones, R.C. (1985) Management of pancreatic trauma. *Am. J. Surg.*, **150**, 698–704
2. Wisner, D.H., Rebekah, L.W. and Frey, C.F. (1990) Diagnosis and treatment of pancreatic injuries; An analysis of management principles. *Arch. Surg.*, **125**, 1109–1113

3. Gorenstein, A., O'Halpin, D. and Wesson, D.E. *et al.* (1987) Blunt injury to the pancreas in children: Selective management based on ultrasound. *J. Pedi. Surg.*, **22**, 1110–1116
4. Karl, H.W. and Chandler, J.G. (1977) Morbidity and mortality of pancreatic injury. *Am. J. Surg.*, **134**, 549–554
5. Northrup, W.F. and Simmons, R.L. (1972) Pancreatic trauma: A review. *Surgery*, **71**, 27–43
6. Bach, R.D. and Frey, C.F. (1971) Diagnosis and treatment of pancreatic trauma. *Am. J. Surg.*, **121**, 20–29
7. Taxier, M., Sivak, M.V. Jr. and Cooperman, A.M. *et al.* (1980) Endoscopic retrograde pancreatography in the evaluation of trauma to the pancreas. *Surg. Gyn. & Obstet.*, **150**, 65–68
8. Laugier, R., Camatte, R. and Sarles, H. (1983) Chronic obstructive pancreatitis after healing of necrotic pseudocyst. *Am. J. Surg.*, **146**, 551–557
9. Turner, L.J. (1983) Chronic pancreatitis and congenital strictures of the pancreatic duct. *Am. J. Surg.*, **145**, 582–589
10. White, T.T. and Kavlic, H. (1973) Congenital obstruction of the pancreatic duct at the duodenum: A report of two cases in adulthood. *Ann. Surg.*, **178**, 194–198
11. Cubilla, A. and Fitzgerald, P.J. (1978) Pancreas cancer I: Duct adenocarcinoma, a clinicopathologic study of 380 patients. *Pathol. Annual.*, **13**, 241–272
12. Sarles, H. (1986) Etiopathogenesis and definition of chronic pancreatitis. *Dig. Dis. and Sci.*, **31**, 91S–107S
13. Singh, S.M. and Reber, H.A. (1990) The pathology of chronic pancreatitis. *World J. Surg.*, **14**, 2–10
14. Nardi, G.L. and Acosta, J.M. (1966) Papillitis as a cause of pancreatitis and abdominal pain. *Ann. Surg.,*, **164**, 611–620
15. Bradley, E.L. III (1982) Pancreatic duct pressure in chronic pancreatitis. *Am. J. Surg.*, **144**, 313–316
16. Austin, J.L., Roberts, C. and Rosenholtz, M.J. *et al.* (1980) Effects of partial duct obstruction and drainage on pancreatic function. *J. Surg. Res.*, **28**, 426–433
17. Barkin, J.S., Ferstenberg, R.M. and Panullo, W. *et al.* (1988) Endoscopic retrograde cholangiopancreatography in pancreatic trauma. *Gastro. Endoscp.*, **34**, 102–105
18. Hayward, S.R., Lucas, C.E. and Sugawa, C. *et al.* (1989) Emergent endoscopic retrograde cholangiopancreatography; a highly specific test for acute pancreatic trauma. *Arch. Surg.*, **124**, 745–746
19. Stone, A., Sugawa, C. and Lucas, C. *et al.* (1990) The role of endoscopic retrograde pancreatography ERP in blunt abdominal trauma. *The Am. Surgeon*, **56**, 715–720

*(Accepted by J.L. Cameron 15 April 1991)*

### INVITED COMMENTARY

### CHRONIC OBSTRUCTIVE PANCREATITIS AS A DELAYED COMPLICATION OF PANCREATIC TRAUMA

E.L. Bradley reports four own patients who developed chronic obstructive pancreatitis proximal to a traumatic pancreatic duct stenosis. It is an important reminder that this condition, although rare, exists and that it needs proper treatment when it occurs. The advent of endoscopic retrograde cholangiopancreaticography (ERCP) has made a correct preoperative diagnosis possible when it comes to the duct stenosis and computerized tomography (CT) helps to visualize the dilated and obstructed distal duct. Thus, the surgical intervention can be based on a careful preoperative mapping. The question arises if it is justified to look for these rare patients by doing a perhaps risky ERCP in the acute phase of a suspected pancreatic trauma? Contrary to Bradley I feel hesitant to such a strategy, especially as it is probable that the duct strictures may take several weeks or months to become manifest. My personal view is that acute ERCP may be of some value if an emergency laparotomy is needed rather than in cases selected for conservative treatment.

As Bradley points out, the incidence of this late complication of pancreatic trauma is not known. However, he speculates that it may become more common with an increasingly conservative attitude to blunt abdominal trauma. This is probably true but still the condition will continue to be rare. Although the pediatric surgeons since long treat most children with pancreatic trauma conservatively or at most with non-operative percutaneous drainage, Bradley in his 20-year collective series could trace only 11 such cases! One explanation of the "relative lack" of these patients might be that some of them are asymptomatic and therefore undiagnosed? In case of acute and complete duct obstruction the obliterated part of the gland will atrophy within a couple of weeks and become "silent" after the initial symptomatic period. Although, perhaps unethical it would be of interest if, let's say 100 individuals with a known major conservatively treated pancreatic trauma in their childhood underwent an ERCP when adult. I think this would present to us a handful who are quite healthy but who harbour a pancreas with an abrupt end!

This nicely written paper is worthwhile as it draws our attention to a rare condition which, if properly managed, can be completely cured.

Ingemar Ihse
Professor of Surgery
Department of Surgery
Lund University
Lund, Sweden

